# Atractylenolide I Ameliorates Acetaminophen-Induced Acute Liver Injury *via* the TLR4/MAPKs/NF-κB Signaling Pathways

**DOI:** 10.3389/fphar.2022.797499

**Published:** 2022-01-21

**Authors:** Zhongyan Du, Zhimei Ma, Shanglei Lai, Qinchao Ding, Ziyi Hu, Wenwen Yang, Qianyu Qian, Linwensi Zhu, Xiaobing Dou, Songtao Li

**Affiliations:** ^1^ School of Basic Medical Sciences, Zhejiang Chinese Medical University, Hangzhou, China; ^2^ School of Life Science, Zhejiang Chinese Medical University, Hangzhou, China; ^3^ School of Animal Science, Zhejiang University, Hangzhou, China; ^4^ Department of Gastroenterology, The First Affiliated Hospital of Zhejiang Chinese Medical University, Hangzhou, China; ^5^ School of Public Health, Zhejiang Chinese Medical University, Hangzhou, China

**Keywords:** atractylenolide I, acetaminophen, liver injury, TLR4/MAPKs/NF-κB, inflammation

## Abstract

**Background:** Acetaminophen (APAP) overdose results in the production of reactive oxygen species (ROS), induces hepatocyte necrosis, and leads to acute liver failure. Atractylenolide I (AO-I), a phytochemical found in *Atractylodes macrocephala* Koidz, is known to exhibit antioxidant activity. However, its clinical benefits against drug-induced liver injury remain largely unclear.

**Purpose:** This study aimed at evaluating the protective effects of AO-I against APAP-induced acute liver injury.

**Methods:** C57BL/6 mice were administered 500 mg/kg APAP to induce hepatotoxicity. AO-Ⅰ (60 and 120 mg/kg) was intragastrically administered 2 h before APAP dosing. Liver histopathological changes, oxidative stress and hepatic inflammation markers from each group were observed.

**Results:** We observed that AO-I treatment significantly reversed APAP-induced liver injury, as evidenced by improved plasma alanine transaminase (ALT) level, aspartate aminotransferase (AST) and liver H&E stain. APAP treatment increased liver malondialdehyde (MDA) content and reduced catalase (CAT) and glutathione (GSH) level; however, these effects were alleviated by AO-I intervention. Moreover, AO-I treatment significantly inhibited APAP-induced activation of pro-inflammatory factors, such as IL-1β, IL-6, and TNF-α, at both the mRNA and protein levels. Mechanistic studies revealed that AO-I attenuated APAP-induced activation of TLR4, NF-κB and MAPKs (including JNK and p38).

**Conclusion:** AO-I mediates protective effects against APAP-induced hepatotoxicity *via* the TLR4/MAPKs/NF-κB pathways. Thus, AO-I is a candidate therapeutic compound for APAP-induced hepatotoxicity.

## Introduction

Acetaminophen (APAP) is one of the most popular antipyretic and analgesic (Lee 2017). The conventional treatment dose is relatively safe for use in humans, but long-term or excessive use of APAP can cause acute liver injury and may lead to death (McGill and Jaeschke 2021). APAP overdose results in the overproduction of reactive free radicals and n-acetyl-p-benzoquinoneimine (NAPQI) (Masubuchi et al., 2005). NAPQI rapidly consumes GSH inside cells and forms complexes with bioactive molecules, which can induce oxidative stress, cause mitochondrial damage, and eventually lead to liver cell damage (Yan et al., 2018).

The therapeutic options for treating APAP-induced hepatotoxicity are currently limited. In recent years, some traditional Chinese medicines and medical herb extracts have been identified to be effective for the prevention and treatment of APAP overdose (Lv et al., 2020; Yang et al., 2020). Thus, It is necessary to explore and develop traditional Chinese medicines in China, in order to obtain safer and effective therapeutic products for the prevention and treatment of APAP-induced acute hepatic injury. Atractylenolide I (AO-I) is a phytochemical found in *Atractylodes macrocephala* Koidz (Fu et al., 2018). AO-I exhibits several pharmacological activities, such as hepatoprotective effects against immunological liver injury, gastrointestinal mucosal injury, and improvement of kidney and lung function (Guo et al., 2021; Song et al., 2017; Wang et al., 2012; Zhang et al., 2015). However, the efficacy of AO-I for treating APAP remains unknown. Therefore, in the present study, we evaluated the protective activity of AO-I on APAP-induced liver injury in mice and explored the potential underlying mechanisms.

## Materials and Methods

### Chemicals

AO-Ⅰ was purchased from Chengdu Ruifensi Biotechnology Co., Ltd. (Chengdu, China, CAS: 73069-13-3). APAP was purchased from Shanghai Wanjiang Biotechnology Co., Ltd. (Shanghai, China, CAS: 103-90-2). NAC was obtained from Sigma-Aldrich (St. Louis, MO, United States, CAS: A7250).

### Animals Experiments

Animal care and experiments complied with National Institutes of Animal Health guidelines on animal research. Male C57BL/6 mice were purchased from Shanghai SLAC laboratory animal Co., Ltd. (Beijing, China). Mice (8 weeks old) were housed at 22 ± 2°C in a 12-h h light/dark cycle in an animal research center at Zhejiang Chinese Medical University (Hangzhou, China). Animals were allocated into the following groups (3–6 mice each): APAP alone treated, APAP and 60 mg/kg AO-I treated, APAP and 120 mg/kg AO-I treated, and APAP and 120 mg/kg NAC treated. The mice were fasted for 12 h with free access to water, and subsequently, intraperitoneally injected with APAP (500 mg/kg) to induce hepatotoxicity. AO-Ⅰ (60 or 120 mg/kg) or NAC (120 mg/kg) was intragastrically administered 2 h before APAP administration. The mice were sacrificed at 8 h and their livers and serum samples were pooled. A portion of the liver was fixed in 4% formaldehyde, and the remaining liver tissue was flash frozen in liquid nitrogen and stored at −80°C until subsequent analyses.

### Measurement of Plasma ALT and AST Levels

Blood samples were collected from the inferior vena cava and centrifuged at 3,000 rpm for 10 min. The plasma was collected as the supernatant and subjected to ALT and AST analysis on an automatic biochemical analyzer using commercial kits (Ningbo MedicalSystem BioTech. Co. Ltd., Ningbo, China).

### Histology

Liver tissues were fixed in 4% formaldehyde, embedded in paraffin, sectioned (5 µm), and subjected to hematoxylin and eosin (H&E) staining (Pi et al., 2021). Sections were examined and imaged using a digital pathology scanner (model: VS120-S6-W, OLYMPUS, Japan). Histological severity of the liver damage was graded using Suzuki’s score criteria on a scale from 0 to 4 (Suzuki et al., 1993).

### Measurement of Liver MDA, CAT, and GSH Levels

For biochemical analysis, approximately 10–20 mg liver tissue was homogenized in 1:9 (w/v) cold physiological saline. The MDA, CAT and GSH levels were determined using commercial kits (Nanjing Jiancheng BioTech. Co. Ltd., Nanjing, China) according to the manufacturer’s instructions.

### RNA Extraction and Real-Time Quantitative PCR

Total RNA was extracted from the liver tissues using Trizol reagent (Sangon Biotech Co., Ltd., Shanghai, China) according to the manufacturer’s instructions. The RNA quality was determined using NanoDrop (Thermo Fisher Scientific, Waltham, MA, United States). Real time PCR was performed using the iQ-SYBR Green PCR Supermix (Bio-Rad, Hercules, CA, United States) on a StepOnePlus real-time PCR system (Thermo Fisher Scientific, Waltham, MA, United States). The primer sequences are listed in Table 1. Relative gene expression was calculated using the 2^−(△△CT)^ analytical method with *18S* as the internal reference gene.

**TABLE 1:** Primer sequence for quantitative real-time PCR.

Gene		Primer sequence (5′–3′)
*TNFA*	Forward	CCC​TCA​CAC​TCA​GAT​CAT​CTT​C
	Reverse	GTT​GGT​TGT​CTT​TGA​GAT​CCA​T
*IL-1B*	Forward	GAA​ATG​CCA​CCT​TTT​GAC​AGT​G
	Reverse	TGG​ATG​CTC​TCA​TCA​GGA​CAG
*IL-6*	Forward	CTC​TGG​GAA​ATC​GTG​GAA​AT
	Reverse	CCA​GTT​TGG​TAG​CAT​CCA​TC
*18S*	Forward	GAA​TGG​GGT​TCA​ACG​GGT​TA
	Reverse	AGG​TCT​GTG​ATG​CCC​TTA​GA

### Western Blotting

Western blotting was performed as described previously (Li et al., 2014) using the following antibodies: anti-TLR4 (Santa Cruz Biotechnology, Dallas, TX, United States); anti-NF-κB, anti-Lamin B, anti-MyD88, anti-JNK, anti-phospho-JNK, anti-p38, anti-phospho-p38, anti-NRF2, anti-HO-1 (Cell Signaling Technology, Danvers, MA, United States); anti-TNFα and anti-IL-1β (Abcam, Cambridge, United Kingdom); anti-IL-6 (Proteintech, Rosemont, IL, United States); anti-GAPDH (Boster Biological Technology, Pleasanton, CA, United States). GAPDH and Lamin B were used as loading controls. Blots were developed using the appropriate HRP-conjugated secondary antibody and ECL kit (Nanjing Vazyme Biotech Co. Ltd., Nanjing, China).

### Statistical Analysis

Statistical analysis was performed using the SPSS statistical software (SPSS V.22.0, SPSS Inc., Chicago, Illinois, United States). One-way ANOVA followed by the least significant difference (LSD) post hoc test was used. Differences between groups were considered statistically significant at *p* < 0.05.

## Results

### AO-I Protects Against APAP-Induced Liver Injury

**FIGURE 1:**
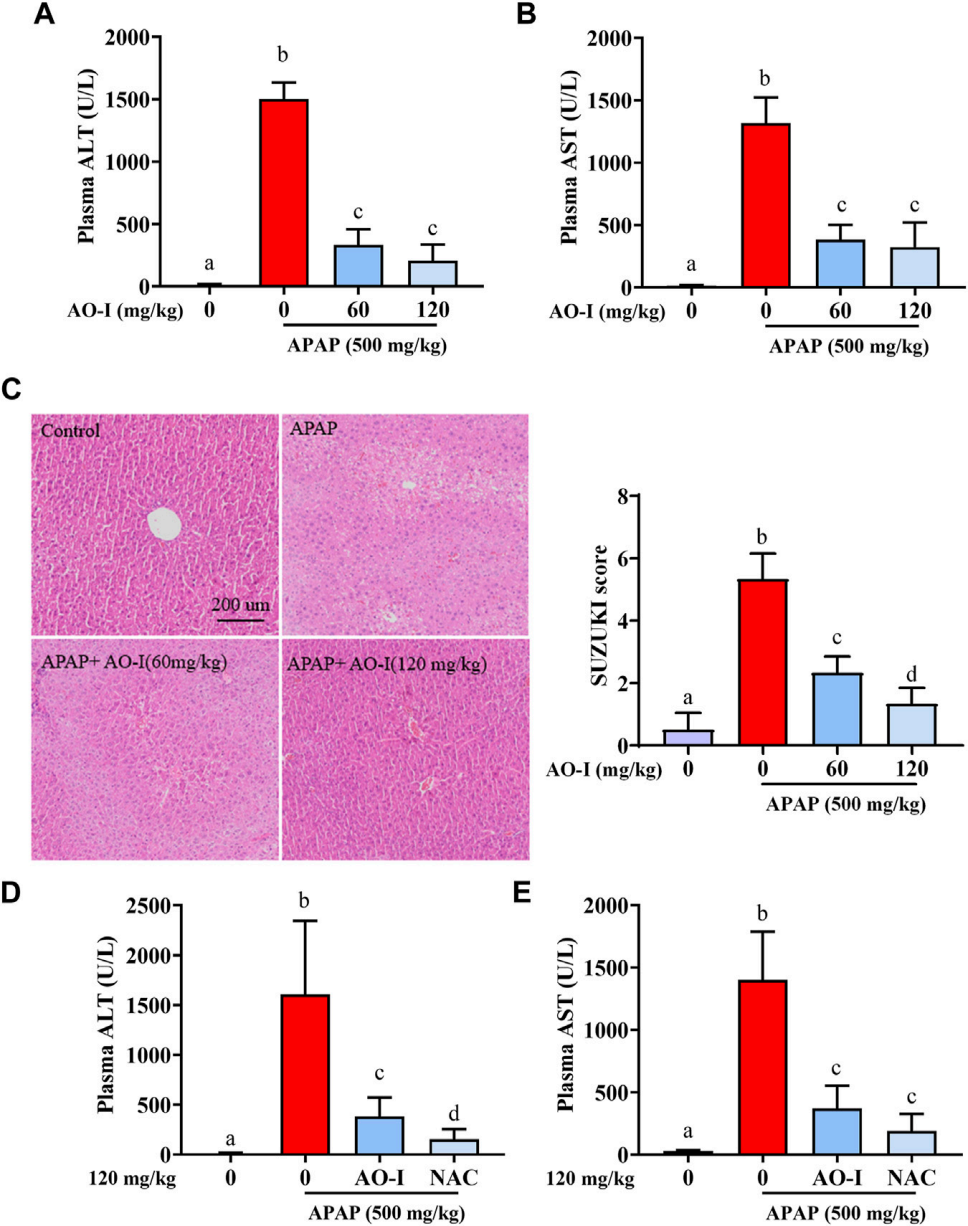
AO-I protects against APAP-induced liver injury. Mice were given AO-I (60 and 120 mg/kg) or NAC (120 mg/kg) *via* intragastric administration 2 h before intraperitoneal APAP administration (500 mg/kg) (*n* = 4–6 in each group). **(A,D)** Plasma ALT level. **(B,E)** Plasma AST level. **(C)** Liver H&E staining (left panel) and Suzuki score (right panel). All data are presented as the mean ± SEM. Bars with different characters present statistically significant results, *p* < 0.05.

To determine whether AO-I attenuates APAP-mediated damage *in vivo*, C57BL/6 mice were administered a single dose of APAP (500 mg/kg). Liver injury was determined by measuring plasma ALT levels and observing histological features after 8 h. There was a significant increase in plasma ALT and AST levels 8 h after APAP administration (*p* < 0.05). Moreover, centrilobular hepatocellular necrosis was observed in livers of mice administered with APAP, as demonstrated by histopathological analysis of H&E-stained liver sections and Suzuki’s scores. In contrast, intragastric administration of AO-I (60 and 120 mg/kg) significantly attenuated the increase in plasma ALT and AST levels, and alleviated APAP-induced hepatotoxicity (Figures 1A–C). These results suggest that AO-I potentially protects against APAP-induced acute hepatic injury.

Current treatment for APAP intoxication is administration of NAC (Yin et al., 2010). We compared protected effect between AO-I and NAC, and observed that AO-I (120 mg/kg, intragastric administration) pretreatment could reduce 76% APAP-induced ALT increase, while the same dose of NAC treatment could reduce 90% APAP-induced ALT increase (Figures 1D,E). Those results indicated that NAC has slightly better protective effect than AO-I. However, AO-I may still be an alternative drug to prevent APAP induced liver injury, especially for people with adverse reactions after taking NAC (18% of patients receiving IV NAC reported anaphylactic reactions, including rash, hypotension, wheezing, and shortness of breath) (Yarema et al., 2018).

### AO-I Ameliorates APAP-Induced Oxidative Injury

Oxidative damage is one of the main characteristics of APAP-induced acute hepatic injury (Wang et al., 2016). Therefore, we investigated whether AO-I protected against oxidative stress-induced hepatotoxicity. We measured MDA and GSH levels, and CAT activity in liver tissues. Our results suggest that APAP administration significantly promoted MDA synthesis, increased GSH levels, and reduced CAT activity in mice livers (*p* < 0.05). In contrast, pretreatment with AO-I markedly blocked these effects (Table 2), and a high dose of AO-I (120 mg/kg) was more efficacious.

**TABLE 2:** AO-I ameliorates APAP-induced oxidative injury.

Group	MDA (nmol/mgprot)	CAT(U/gprot)	GSH(μmol/L)
Control	6.10 ± 1.01^a^	547.40 ± 45.19^a^	103.29 ± 32.69^a^
APAP (500 mg/kg)	20.07 ± 0.86^b^	341.50 ± 59.75^b^	5.62 ± 0.37^b^
APAP (500 mg/kg)+AO-Ⅰ(60 mg/kg)	9.22 ± 1.28^c^	422.05 ± 35.80^c^	13.57 ± 1.75^c^
APAP (500 mg/kg)+AO-Ⅰ(120 mg/kg)	4.63 ± 0.77^a^	510.12 ± 52.40^a^	22.89 ± 7.65^c^

Liver tissues were obtained from the mice 8 h after APAP, challenge for the measurement of MDA, content; CAT activity, and GSH, level. All data are presented as the mean ± SEM., bars with different characters present statistically significant results, *p* < 0.05.

### AO-I Inhibits the Expression of Inflammatory Factors Induced by APAP Treatment

**FIGURE 2:**
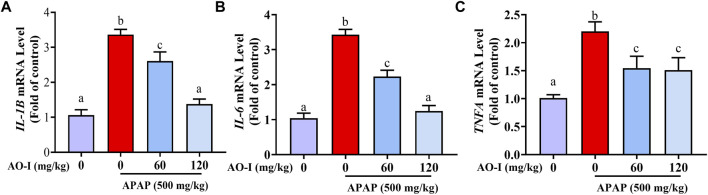
AO-I inhibits APAP-induced gene expression of inflammatory factors. Gene expression level of **(A)** *IL-1B*, **(B)** *IL-6* and **(C)** *TNFA*. All data are presented as the mean ± SEM. Bars with different characters present statistically significant results, *p* < 0.05.

Since APAP-induced hepatotoxicity is associated with increased inflammation (Liu et al., 2004), we analyzed APAP-induced inflammatory response by assessing the expression of *IL-1B*, *IL-6*, and *TNFA* in the livers of APAP-administrated mice treated with or without AO-I. We found that the mRNA expression of pro-inflammatory cytokines *IL-1B*, *IL-6*, and *TNFA* significantly increased in the APAP-only treated group (*p* < 0.05). In contrast, AO-I treatment reduced the expression of these pro-inflammatory factors (Figures 2A–C).

### AO-I Regulates the NF-κB Signaling Pathway

**FIGURE 3:**
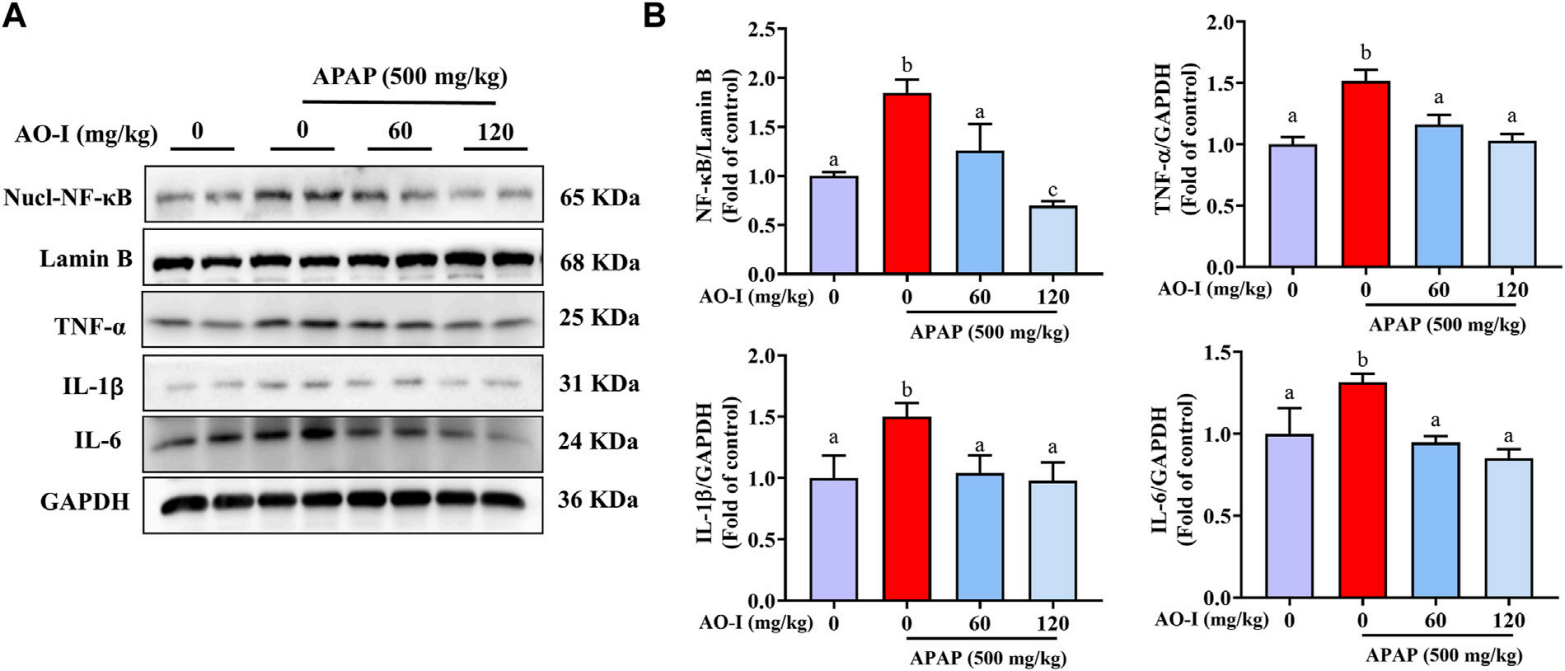
AO-I inhibits APAP-induced NF-κB signaling pathway. **(A)** Effect of AO-I on APAP-induced nuclear translocation of NF-κB, IL-1β, IL-6, and TNF-α in the liver. **(B)** Quantitative map of NF-κB, IL-1β, IL-6, and TNF-α protein expression. All data are presented as the mean ± SEM. Bars with different characters present statistically significant results, *p* < 0.05.

We measured the protein expression of the abovementioned pro-inflammatory factors, and the results were consistent with those of the mRNA expression (Figures 3A,B). In addition, we analyzed the expression of NF-κB, a master transcription factor involved in immune system functioning (Luo et al., 2005). APAP administration induced NF-κB expression in the nuclei of treated cells. However, AO-I treatment (60 and 120 mg/kg) significantly inhibited the nuclear translocation of NF- κB (*p* < 0.05). Taken together, these results suggest that AO-I attenuates APAP-induced liver inflammation by regulating NF-κB.

### AO-I Regulates the TLR4 Signaling Pathway

**FIGURE 4:**
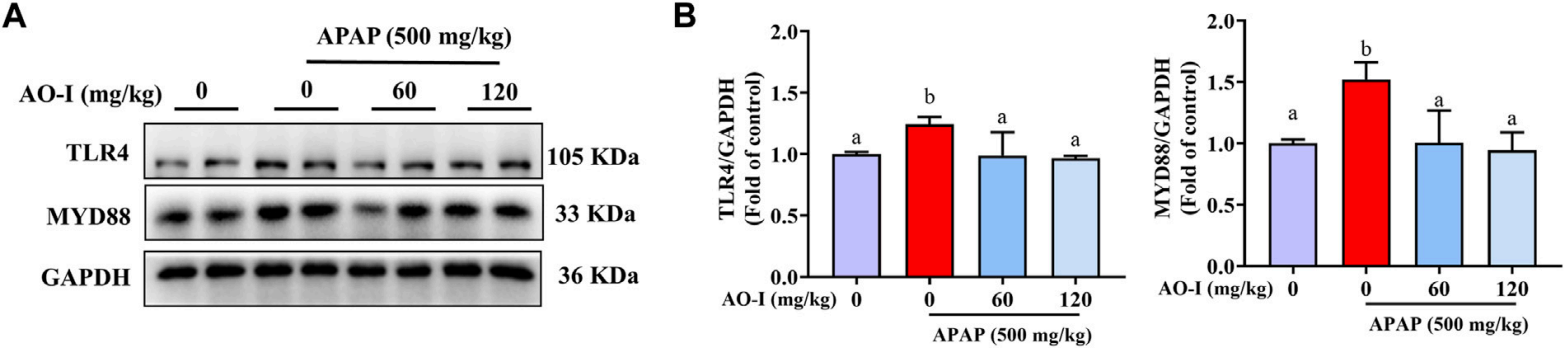
AO-I inhibits APAP-induced TLR4 signaling pathway. **(A)** Effects of AO-I on APAP-induced TLR4 and MYD88 expression in the liver. **(B)** Quantitative map of TLR4 and MYD88 protein expression. All data are presented as the mean ± SEM. Bars with different characters present statistically significant results, *p* < 0.05.

Activation of TLR4 is closely associated with the expression of inflammatory factors involved in mediating liver injury in APAP-treated mice (Xu et al., 2020). MyD88 is a key molecule acting downstream of TLR4 (Płóciennikowska et al., 2015). Our data demonstrated that APAP administration increased TLR4 and MYD88 expression; however, AO-I pretreatment (60 and 120 mg/kg) significantly reversed these effects (*p* < 0.05, Figures 4A,B), indicating that AO-I prevents APAP-induced inflammatory reaction partly *via* inhibiting the NF-κB pathway.

### AO-I Regulates the MAPK Signaling Pathway

**FIGURE 5:**
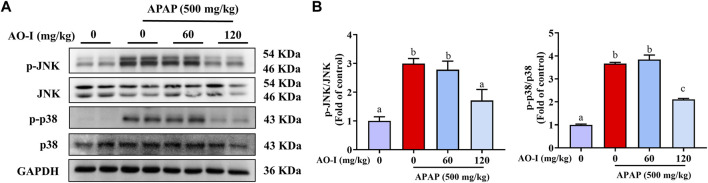
AO-I inhibits APAP-induced MAPK signaling pathway. **(A)** Effects of AO-I on APAP-induced p-JNK and p-p38 expression in the liver. **(B)** Quantitative map of p-JNK and p-p38 protein expression. All data are presented as the mean ± SEM. Bars with different characters present statistically significant results, *p* < 0.05.

We measured the expression of proteins involved in the mitogen-activated protein kinase (MAPK) signaling pathway in our study to explore whether AO-I regulates MAPK pathway to alleviate APAP-induced liver injury. Western blot analysis for evaluating the expression of JNK, phosphorylated (p)-JNK, p38 and p-p38 proteins indicated that APAP treatment increased the activation of JNK and p38, i.e., the expression of p-JNK and p-p38, respectively. Compared with APAP-alone treated mice, AO-I treated mice exhibited reduced expression of p-JNK and p-p38 (Figures 5A,B). These findings illustrate that MAPK signaling is involved in exerting the protective effects of AO-I in APAP-induced liver injury.

## Discussion

In the present study, we have shown that AO-I, a phytochemical found in *A. macrocephala* Koidz*.*, protects against APAP-induced hepatotoxicity in acute hepatic injury. We also provide evidence that these beneficial effects partly through the NF-κB and MAPK signaling pathways.

Natural products have made significant contributions to drug discovery as they offer several potential advantages over conventional chemical-based drugs. Several studies have reported that plant extracts or pure compounds can reduce APAP-induced hepatic injury (Leng et al., 2018; Zai et al., 2018; Lv et al., 2020; Yang et al., 2020). AO-I, extracted from *A. macrocephala* Koidz., has been reported to exert protective effects against liver diseases (Wang et al., 2012). AO-I is widely used in various fields and has various prospective useful applications (Tang et al., 2017; Wang et al., 2019a; Zhu et al., 2020). The anti-inflammatory and antioxidant effects of AO-I are well-documented (Li et al., 2007; Zhang et al., 2015). Thus, in this study, we sought to investigate the potential role of AO-I in alleviating APAP-induced acute hepatic injury *in vivo*, which remain largely unknown. We administered different doses of AO-I before APAP administration, and analyzed liver histology, and plasma ALT and AST levels to determine hepatocellular toxicity following APAP injection. We found that hepatocytes are extremely sensitive to APAP stimulation, as evidenced by high plasma ALT and AST levels and pathological changes observed in the livers of APAP-treated mice. Thus, our results showed that AO-I could significantly reduce APAP-induced acute hepatic injury in mice in a dose-dependent manner.

APAP overdose-induced hepatotoxicity is the most common cause of acute-liver failure worldwide (Larson 2007). The APAP overdose-induced fatal hepatotoxicity is characterized by multiple indicators of cell damage activated by oxidative stress and endoplasmic reticulum stress (Zhang et al., 2016). Excessive oxidative stress leads to cell death and lipid peroxidation and destroys cellular components (Li et al., 2015). MDA, an aldehyde produced in the process of free radical-induced lipid peroxidation, can reflect the degree of lipid peroxidation in the body and is a marker of oxidative stress (Yamada et al., 2020; Zheng et al., 2019). CAT is a vital oxidoreductase, which catalyzes the decomposition of hydrogen peroxide into oxygen and water (Zhao et al., 2019). Thus, CAT protects cells from oxidative stress. Hepatic GSH is one of the key enzymes involved in the detoxification of NAPQI, the metabolic product of APAP (He et al., 2017). Our results demonstrate that APAP administration significantly reduced CAT and GSH levels, and increased MDA levels. Thus, APAP dysregulates redox balance and promotes reactive oxygen species (ROS) production, leading to lipid peroxidation (Zhong et al., 2021). However, after pretreatment with AO-I, the MDA levels were normalized, and the CAT and GSH levels were restored in the liver. These results suggest that AO-I intervention reduces ROS production to protect against APAP-induced hepatotoxicity.

APAP-induced acute liver injury induces the expression of proinflammatory factors (such as IL-1β, IL-6, and TNF-α), which can then exacerbate organ damage, especially cause hepatocyte injury to damage the liver (Chen et al., 2020; Raevens et al., 2020; Saad et al., 2020; Chen et al., 2021). Therefore, inflammatory response is considered one of the basic mechanisms of APAP-induced liver injury (Huang et al., 2013). Furthermore, NF-κB induces the expressions of various proinflammatory factors that play important pathological roles in the liver (Wang et al., 2019b). APAP-induced hepatotoxicity may result in the nuclear translocation of NF-κB (p65). Activation of NF-κB induces the transcription of some inflammatory genes, including *IL-1B*, *IL-6*, and *TNFA* (Kawai and Akira 2007). Our results showed that the mRNA expression level of *IL-1B*, *IL-6*, and *TNFA* was significantly increased in the liver, and the results of protein expression level were consistent with these findings. In contrast, AO-I pretreatment inhibited the release of these proinflammatory cytokines following APAP administration. Moreover, AO-I prevented APAP-induced nuclear translocation of NF-κB. It, therefore, seems that the inhibitory effect on the expression of the NF-κB pathway may be the result of the anti-inflammatory effects of AO-1.

TLR4 activation can induce innate immune response and activate the NF-κB signaling pathway, thereby leading to the release of various proinflammatory cytokines and a systemic inflammatory response (Lawrence 2009; Takeda and Akira 2004). To further elucidate the molecular mechanism underlying the protective effects of AO-I against APAP-induced hepatotoxicity, we examined the TLR4 signaling pathway and the expression of its downstream protein factor MYD88. We found that AO-I significantly down-regulated the expression of TLR4 and MYD88. MYD88 dependent pathway is one of the main branches of the TLR4 signaling pathway. The subsequent TLR4 signaling cascade can be summarized as follows: TLR4 binds explicitly to the MYD88 adaptor molecule, triggering a series of interlocking reactions, resulting in the activation of downstream tumor necrosis factor receptor-related factor (TRAF6) (Cha et al., 2018), which in turn can induce two different signal transduction pathways: MAPK and NF-κB signaling pathway. MAPKs (including p38, ERK, and JNK) play an essential role in liver injury, oxidative stress, and apoptosis. Activation of JNK and p38 leads to mitochondrial dysfunction, which in turn induces hepatocyte apoptosis in APAP-induced liver injury (Tashiro et al., 2021). We found similar findings in the present study; APAP significantly increased the phosphorylation of JNK and p38. After AO-I intervention, phosphorylation levels of JNK and p38 were significantly downregulated. Taken together, these data suggest that the protective effect of AO-I in suppressing APAP-induced liver injury may be mediated *via* regulation of the TLR4/MAPK/NF-κB signaling pathways.

In summary, the present study demonstrated that AO-I exerts a potential therapeutic effect against APAP-induced acute liver injury, which can be attributed to its anti-inflammatory and anti-oxidative properties. The therapeutic efficacy of AO-I against APAP-induced liver injury may be due to the regulation of the TLR4/MAPK/NF-κB pathways. Our study outcomes provide essential insights into the mechanisms by which AO-I treatment confers protection against hepatotoxicity. Moreover, our findings suggest that AO-I may be as a potential therapeutic agent for APAP-induced acute liver injury.

## Data Availability

The original contributions presented in the study are included in the article/Supplementary Material, further inquiries can be directed to the corresponding authors.

## References

[B1] Cha H. Lee S. Lee J. H. Park J. W. (2018). Protective Effects of P-Coumaric Acid against Acetaminophen-Induced Hepatotoxicity in Mice. Food Chem. Toxicol. 121, 131–139. 10.1016/j.fct.2018.08.060 30149109

[B2] Chen W. Fan J. Zai W. Zhang X. Zeng X. Luan J. (2020). Interleukin-22 Drives a Metabolic Adaptive Reprogramming to Maintain Mitochondrial Fitness and Treat Liver Injury. Theranostics 10 (13), 5879–5894. 10.7150/thno.43894 32483425 PMC7254999

[B3] Chen W. Shen Y. Fan J. Zeng X. Zhang X. Luan J. (2021). IL-22-mediated Renal Metabolic Reprogramming via PFKFB3 to Treat Kidney Injury. Clin. Transl Med. 11 (2), e324. 10.1002/ctm2.324 33634980 PMC7901723

[B4] Fu X. Q. Chou J. Y. Li T. Zhu P. L. Li J. K. Yin C. L. (2018). The JAK2/STAT3 Pathway Is Involved in the Anti-melanoma Effects of Atractylenolide I. Exp. Dermatol. 27 (2), 201–204. 10.1111/exd.13454 29078004

[B5] Guo Y. Xiao Y. Zhu H. Guo H. Zhou Y. Shentu Y. (2021). Inhibition of Proliferation-Linked Signaling Cascades with Atractylenolide I Reduces Myofibroblastic Phenotype and Renal Fibrosis. Biochem. Pharmacol. 183, 114344. 10.1016/j.bcp.2020.114344 33221275

[B6] He Y. Feng D. Li M. Gao Y. Ramirez T. Cao H. (2017). Hepatic Mitochondrial DNA/Toll-like Receptor 9/MicroRNA-223 Forms a Negative Feedback Loop to Limit Neutrophil Overactivation and Acetaminophen Hepatotoxicity in Mice. Hepatology 66 (1), 220–234. 10.1002/hep.29153 28295449 PMC5481471

[B7] Huang G. J. Deng J. S. Huang S. S. Lee C. Y. Hou W. C. Wang S. Y. (2013). Hepatoprotective Effects of Eburicoic Acid and Dehydroeburicoic Acid from Antrodia Camphorata in a Mouse Model of Acute Hepatic Injury. Food Chem. 141 (3), 3020–3027. 10.1016/j.foodchem.2013.03.061 23871054

[B8] Kawai T. Akira S. (2007). Signaling to NF-kappaB by Toll-like Receptors. Trends Mol. Med. 13 (11), 460–469. 10.1016/j.molmed.2007.09.002 18029230

[B9] Larson A. M. (2007). Acetaminophen hepatotoxicityClinics in Liver Disease. Clin. Liver Dis. 11 (3), 525–vi. 10.1016/j.cld.2007.06.006 17723918

[B10] Lawrence T. (2009). The Nuclear Factor NF-kappaB Pathway in Inflammation. Cold Spring Harb Perspect. Biol. 1 (6), a001651. Epub 2010/05/12. PubMed PMID: 20457564. 10.1101/cshperspect.a001651 20457564 PMC2882124

[B11] Lee W. M. (2017). Acetaminophen (APAP) Hepatotoxicity-Isn't it Time for APAP to Go Away. J. Hepatol. 67 (6), 1324–1331. 10.1016/j.jhep.2017.07.005 28734939 PMC5696016

[B12] Leng J. Wang Z. Fu C. L. Zhang J. Ren S. Hu J. N. (2018). NF-κB and AMPK/PI3K/Akt Signaling Pathways Are Involved in the Protective Effects of Platycodon Grandiflorum Saponins against Acetaminophen-Induced Acute Hepatotoxicity in Mice. Phytother Res. 32 (11), 2235–2246. 10.1002/ptr.6160 30039882

[B13] Li C. Q. He L. C. Jin J. Q. (2007). Atractylenolide I and Atractylenolide III Inhibit Lipopolysaccharide-Induced TNF-Alpha and NO Production in Macrophages. Phytother Res. 21 (4), 347–353. 10.1002/ptr.2040 17221938

[B14] Li S. Li J. Shen C. Zhang X. Sun S. Cho M. (2014). tert-Butylhydroquinone (tBHQ) Protects Hepatocytes against Lipotoxicity via Inducing Autophagy Independently of Nrf2 Activation. Biochim. Biophys. Acta 1841 (1), 22–33. 10.1016/j.bbalip.2013.09.004 24055888 PMC3884638

[B15] Li Y. Y. Huang S. S. Lee M. M. Deng J. S. Huang G. J. (2015). Anti-inflammatory Activities of Cardamonin from Alpinia Katsumadai through Heme Oxygenase-1 Induction and Inhibition of NF-Κb and MAPK Signaling Pathway in the Carrageenan-Induced Paw Edema. Int. Immunopharmacol 25 (2), 332–339. 10.1016/j.intimp.2015.02.002 25681284

[B16] Liu Z. X. Govindarajan S. Kaplowitz N. (2004). Innate Immune System Plays a Critical Role in Determining the Progression and Severity of Acetaminophen Hepatotoxicity. Gastroenterology 127 (6), 1760–1774. 10.1053/j.gastro.2004.08.053 15578514

[B17] Luo J. L. Kamata H. Karin M. (2005). IKK/NF-kappaB Signaling: Balancing Life and Death-Aa New Approach to Cancer Therapy. J. Clin. Invest. 115 (10), 2625–2632. 10.1172/JCI26322 16200195 PMC1236696

[B18] Lv H. Zhu C. Wei W. Lv X. Yu Q. Deng X. (2020). Enhanced Keap1-Nrf2/Trx-1 axis by Daphnetin Protects against Oxidative Stress-Driven Hepatotoxicity via Inhibiting ASK1/JNK and Txnip/NLRP3 Inflammasome Activation. Phytomedicine 71, 153241. 10.1016/j.phymed.2020.153241 32454347

[B19] Masubuchi Y. Suda C. Horie T. (2005). Involvement of Mitochondrial Permeability Transition in Acetaminophen-Induced Liver Injury in Mice. J. Hepatol. 42 (1), 110–116. 10.1016/j.jhep.2004.09.015 15629515

[B20] McGill M. R. Jaeschke H. (2021). Biomarkers of Mitotoxicity after Acute Liver Injury: Further Insights into the Interpretation of Glutamate Dehydrogenase. J. Clin. Transl Res. 7 (1), 61–65. 34027202 PMC8132186

[B21] Pi A. Jiang K. Ding Q. Lai S. Yang W. Zhu J. (2021). Alcohol Abstinence Rescues Hepatic Steatosis and Liver Injury via Improving Metabolic Reprogramming in Chronic Alcohol-Fed Mice. Front. Pharmacol. 12, 752148. 10.3389/fphar.2021.752148 34603062 PMC8481816

[B22] Płóciennikowska A. Hromada-Judycka A. Borzęcka K. Kwiatkowska K. (2015). Co-operation of TLR4 and Raft Proteins in LPS-Induced Pro-inflammatory Signaling. Cell Mol. Life Sci. 72 (3), 557–581. 10.1007/s00018-014-1762-5 25332099 PMC4293489

[B23] Raevens S. Van Campenhout S. Debacker P. J. Lefere S. Verhelst X. Geerts A. (2020). Combination of Sivelestat and N-Acetylcysteine Alleviates the Inflammatory Response and Exceeds Standard Treatment for Acetaminophen-Induced Liver Injury. J. Leukoc. Biol. 107 (2), 341–355. 10.1002/JLB.5A1119-279R 31841237

[B24] Saad K. M. Shaker M. E. Shaaban A. A. Abdelrahman R. S. Said E. (2020). The C-Met Inhibitor Capmatinib Alleviates Acetaminophen-Induced Hepatotoxicity. Int. Immunopharmacol 81, 106292. 10.1016/j.intimp.2020.106292 32062076

[B25] Song H. P. Hou X. Q. Li R. Y. Yu R. Li X. Zhou S. N. (2017). Atractylenolide I Stimulates Intestinal Epithelial Repair through Polyamine-Mediated Ca2+ Signaling Pathway. Phytomedicine 28, 27–35. 10.1016/j.phymed.2017.03.001 28478810

[B26] Suzuki S. Toledo-Pereyra L. H. Rodriguez F. J. Cejalvo D. (1993). Neutrophil Infiltration as an Important Factor in Liver Ischemia and Reperfusion Injury. Modulating Effects of FK506 and Cyclosporine. Transplantation 55 (6), 1265–1272. 10.1097/00007890-199306000-00011 7685932

[B27] Takeda K. Akira S. (2004). TLR Signaling Pathways. Semin. Immunol. 16, 3–9. 10.1016/j.smim.2003.10.003 14751757

[B28] Tang X. M. Liao Z. K. Huang Y. W. Lin X. Wu L. C. (2017). Atractylenolide Ⅰ Protects against Lipopolysaccharide-Induced Disseminated Intravascular Coagulation by Anti-inflammatory and Anticoagulation Effect. Asian Pac. J. Trop. Med. 10 (6), 582–587. 10.1016/j.apjtm.2017.06.007 28756923

[B29] Tashiro S. Tanaka M. Goya T. Aoyagi T. Kurokawa M. Imoto K. (2021). Pirfenidone Attenuates Acetaminophen-Induced Liver Injury via Suppressing C-Jun N-Terminal Kinase Phosphorylation. Toxicol. Appl. Pharmacol. 434, 115817. 10.1016/j.taap.2021.115817 34890640

[B30] Wang C. Geng Q. Wang Y. (2012). Protective Effect of Atractylenolide I on Immunological Liver Injury. Zhongguo Zhong Yao Za Zhi 37 (12), 1809–1813. 10.4268/cjcmm20121224 22997829

[B31] Wang Q. Guo W. Hao B. Shi X. Lu Y. Wong C. W. (2016). Mechanistic Study of TRPM2-Ca(2+)-CAMK2-BECN1 Signaling in Oxidative Stress-Induced Autophagy Inhibition. Autophagy 12 (8), 1340–1354. 10.1080/15548627.2016.1187365 27245989 PMC4968236

[B32] Wang M. Hu R. Wang Y. Liu L. You H. Zhang J. (2019a). Atractylenolide III Attenuates Muscle Wasting in Chronic Kidney Disease via the Oxidative Stress-Mediated PI3K/AKT/mTOR Pathway. Oxidative Med. Cell. longevity 2019, 1875471. 10.1155/2019/1875471 PMC6501186 31178951

[B33] Wang Z. Hao W. Hu J. Mi X. Han Y. Ren S. (2019b). Maltol Improves APAP-Induced Hepatotoxicity by Inhibiting Oxidative Stress and Inflammation Response via NF-Κb and PI3K/Akt Signal Pathways. Antioxidants (Basel) 8 (9), 395. 10.3390/antiox8090395 PMC6769439 31547366

[B34] Xu Q. Fan Y. Loor J. J. Liang Y. Sun X. Jia H. (2020). Cardamonin Reduces Acetaminophen-Induced Acute Liver Injury in Mice via Activating Autophagy and NFE2L2 Signaling. Front. Pharmacol. 11, 601716. 10.3389/fphar.2020.601716 33364966 PMC7751642

[B35] Yamada N. Karasawa T. Kimura H. Watanabe S. Komada T. Kamata R. (2020). Ferroptosis Driven by Radical Oxidation of N-6 Polyunsaturated Fatty Acids Mediates Acetaminophen-Induced Acute Liver Failure. Cell Death Dis 11 (2), 144. 10.1038/s41419-020-2334-2 32094346 PMC7039960

[B36] Yan M. Huo Y. Yin S. Hu H. (2018). Mechanisms of Acetaminophen-Induced Liver Injury and its Implications for Therapeutic Interventions. Redox Biol. 17, 274–283. 10.1016/j.redox.2018.04.019 29753208 PMC6006912

[B37] Yang R. Song C. Chen J. Zhou L. Jiang X. Cao X. (2020). Limonin Ameliorates Acetaminophen-Induced Hepatotoxicity by Activating Nrf2 Antioxidative Pathway and Inhibiting NF-Κb Inflammatory Response via Upregulating Sirt1. Phytomedicine 69, 153211. 10.1016/j.phymed.2020.153211 32259676

[B38] Yarema M. Chopra P. Sivilotti M. L. A. Johnson D. Nettel-Aguirre A. Bailey B. (2018). Anaphylactoid Reactions to Intravenous N-Acetylcysteine during Treatment for Acetaminophen Poisoning. J. Med. Toxicol. 14 (2), 120–127. 10.1007/s13181-018-0653-9 29423816 PMC5962465

[B39] Yin H. Cheng L. Holt M. Hail N. Maclaren R. Ju C. (2010). Lactoferrin Protects against Acetaminophen-Induced Liver Injury in Mice. Hepatology 51 (3), 1007–1016. 10.1002/hep.23476 20099297 PMC2908515

[B40] Zai W. Chen W. Luan J. Fan J. Zhang X. Wu Z. (2018). Dihydroquercetin Ameliorated Acetaminophen-Induced Hepatic Cytotoxicity via Activating JAK2/STAT3 Pathway and Autophagy. Appl. Microbiol. Biotechnol. 102 (3), 1443–1453. 10.1007/s00253-017-8686-6 29243082

[B41] Zhang J. L. Huang W. M. Zeng Q. Y. (2015). Atractylenolide I Protects Mice from Lipopolysaccharide-Induced Acute Lung Injury. Eur. J. Pharmacol. 765, 94–99. 10.1016/j.ejphar.2015.08.022 26297303

[B42] Zhang Y. Zhang F. Wang K. Liu G. Yang M. Luan Y. (2016). Protective Effect of Allyl Methyl Disulfide on Acetaminophen-Induced Hepatotoxicity in Mice. Chem. Biol. Interact 249, 71–77. 10.1016/j.cbi.2016.03.008 26969520

[B43] Zhao M. X. Wen J. L. Wang L. Wang X. P. Chen T. S. (2019). Intracellular Catalase Activity Instead of Glutathione Level Dominates the Resistance of Cells to Reactive Oxygen Species. Cell Stress Chaperones 24 (3), 609–619. 10.1007/s12192-019-00993-1 30989612 PMC6527626

[B44] Zheng H. J. Guo J. Jia Q. Huang Y. S. Huang W. J. Zhang W. (2019). The Effect of Probiotic and Synbiotic Supplementation on Biomarkers of Inflammation and Oxidative Stress in Diabetic Patients: a Systematic Review and Meta-Analysis of Randomized Controlled Trials. Pharmacol. Res. 142, 303–313. 10.1016/j.phrs.2019.02.016 30794924

[B45] Zhong X. Zhang Z. Shen H. Xiong Y. Shah Y. M. Liu Y. (2021). Hepatic NF-Κb-Inducing Kinase and Inhibitor of NF-Κb Kinase Subunit α Promote Liver Oxidative Stress, Ferroptosis, and Liver Injury. Hepatol. Commun. 5 (10), 1704–1720. 10.1002/hep4.1757 34558831 PMC8485893

[B46] Zhu C. Zhang L. Liu Z. Li C. Bai Y. Wang L. (2020). Atractylenolide III Reduces NLRP3 Inflammasome Activation and Th1/Th2 Imbalances in Both *In Vitro* and *In Vivo* Models of Asthma. Clin. Exp. Pharmacol. Physiol. 47 (8), 1360–1367. 10.1111/1440-1681.13306 32196713

