# Supplementary material for: Atractylenolide I Ameliorates Acetaminophen-Induced Acute Liver Injury via the TLR4/MAPKs/NF-κB Signaling Pathways
Source: Front Pharmacol. 2022 Jan 21;13:797499. doi: 10.3389/fphar.2022.797499 (PMC8815859; doi:10.3389/fphar.2022.797499)
Supplement: Supplementary file 1 [file DataSheet3.DOCX]

**Supplementary data**

**Figure S1**


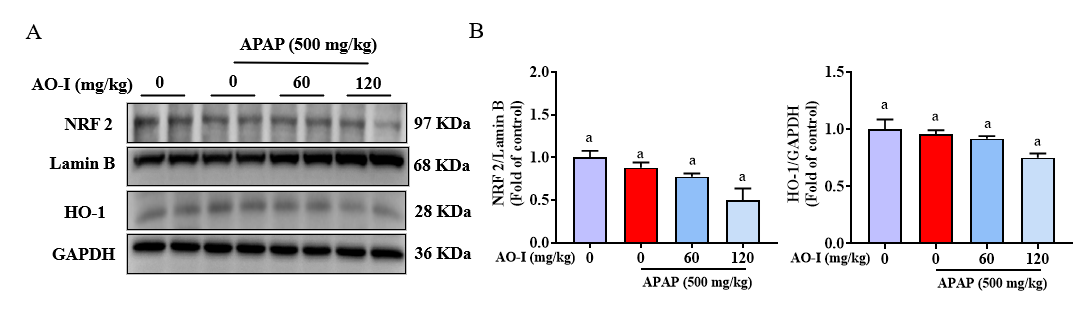


**Figure S1.** Effect of AO-I on APAP-induced NRF2 and HO-1 expression in the liver. (A) Protein levels of NRF2 and its downstream target gene HO-1 were detected by western blot. (B) Quantitative map of NRF2 and HO-1 protein expression. All data are presented as the mean ± SEM. Bars with different characters present statistically significant results, *P* < 0.05.
