# Supplementary figures and images for: Atractylenolide I Ameliorates Acetaminophen-Induced Acute Liver Injury via the TLR4/MAPKs/NF-κB Signaling Pathways
Source: Front Pharmacol. 2022 Jan 21;13:797499. doi: 10.3389/fphar.2022.797499 (PMC8815859; doi:10.3389/fphar.2022.797499)

Control APAP


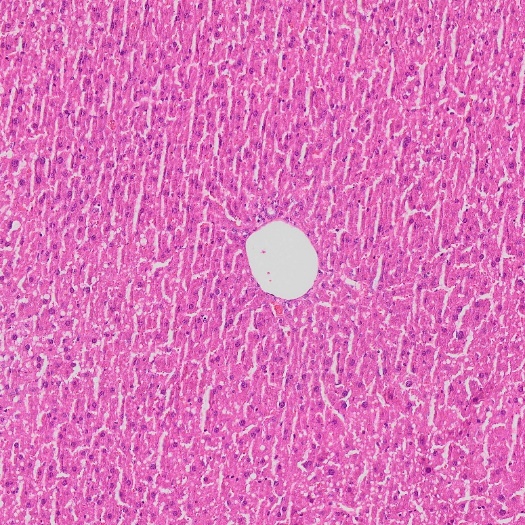

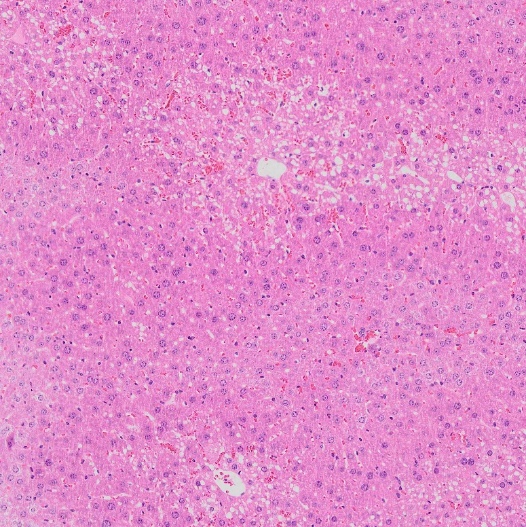


APAP+AO-Ⅰ(60mg/kg) APAP+AO-Ⅰ(120mg/kg)


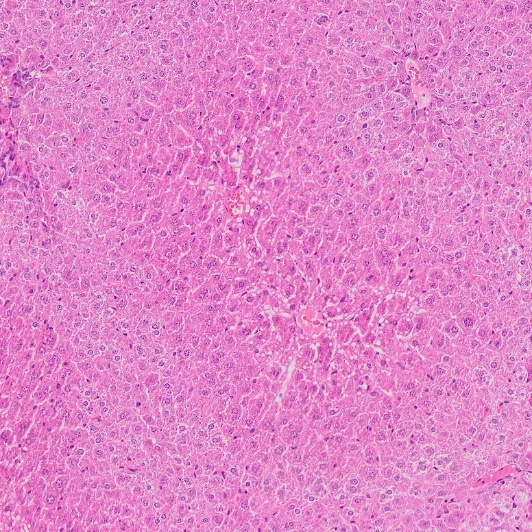

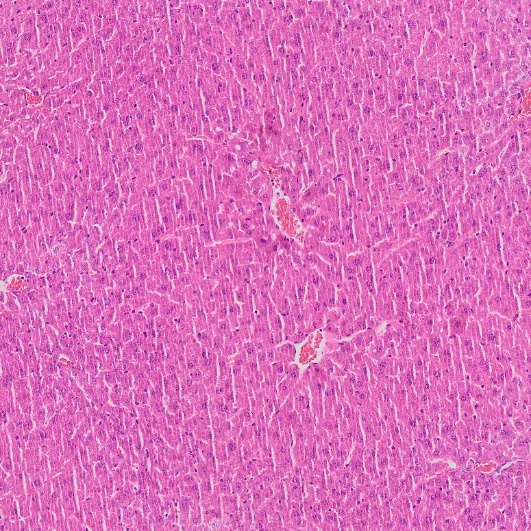


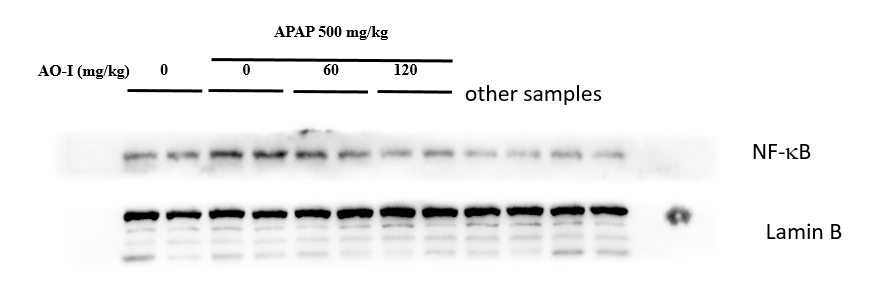

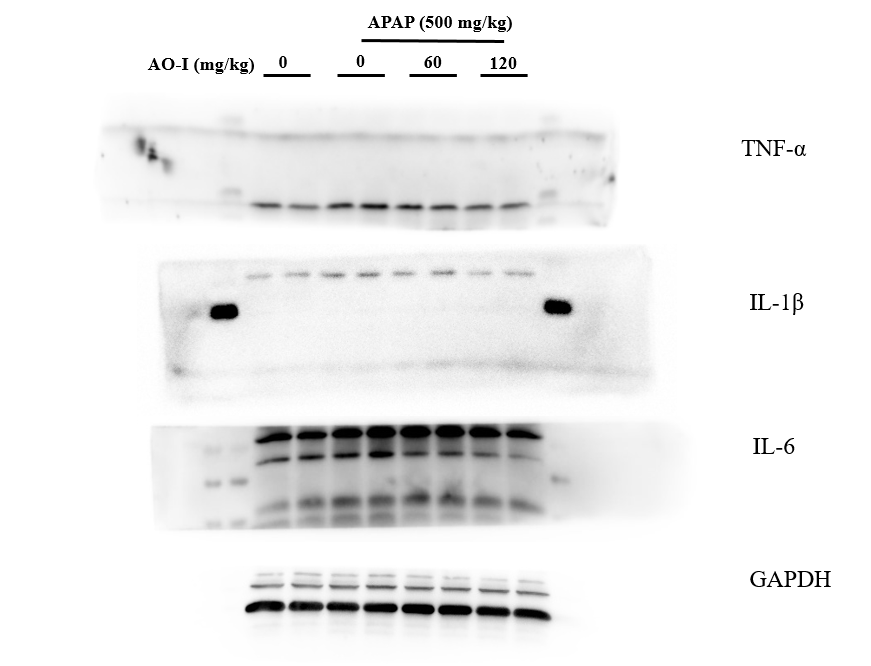

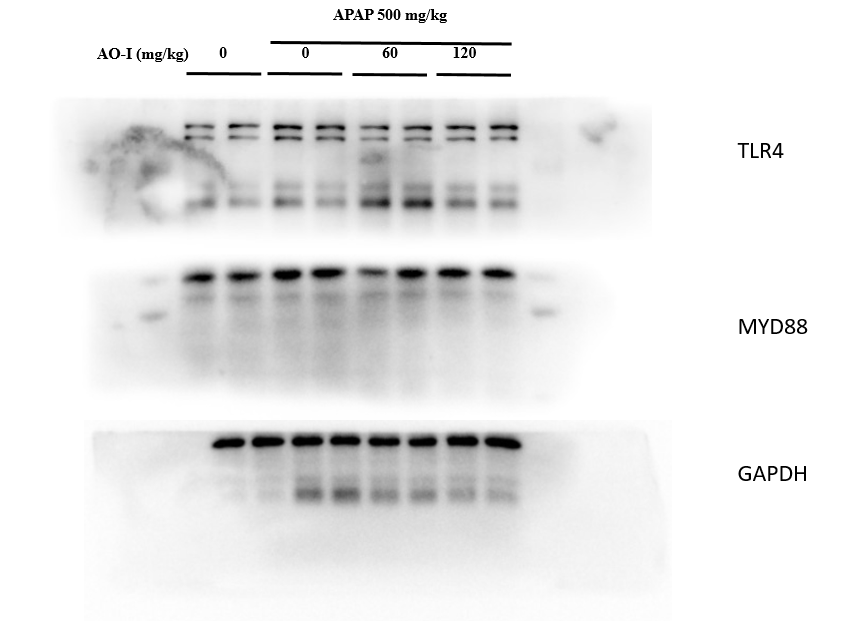

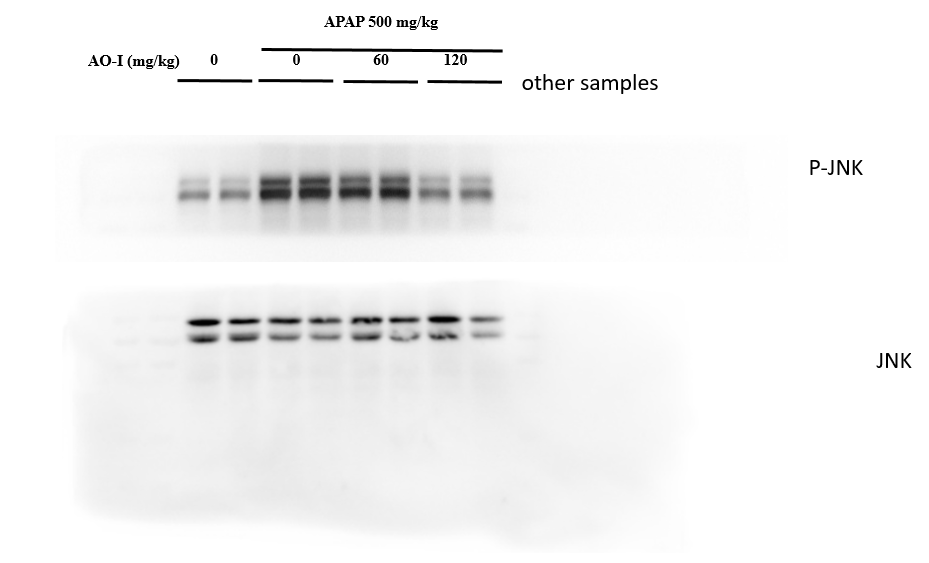

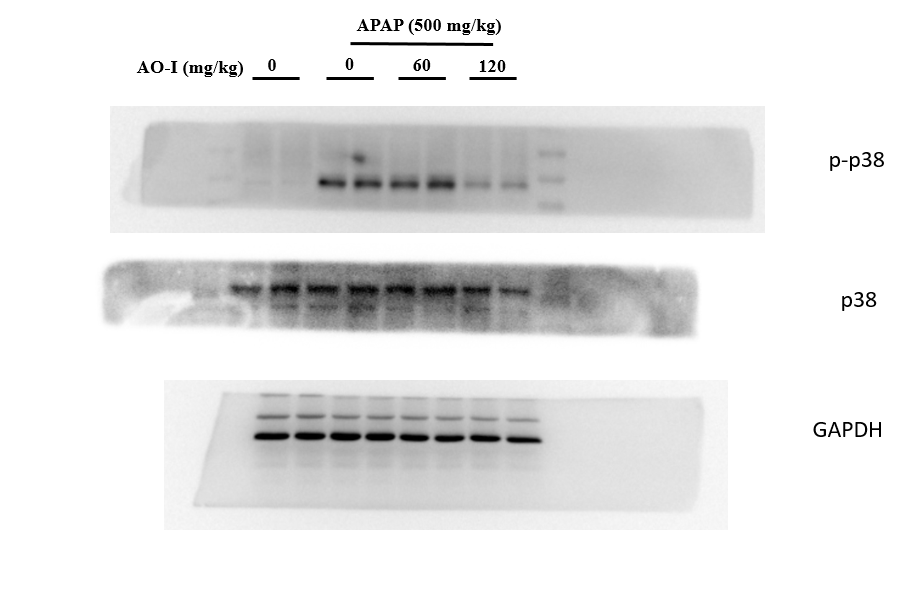

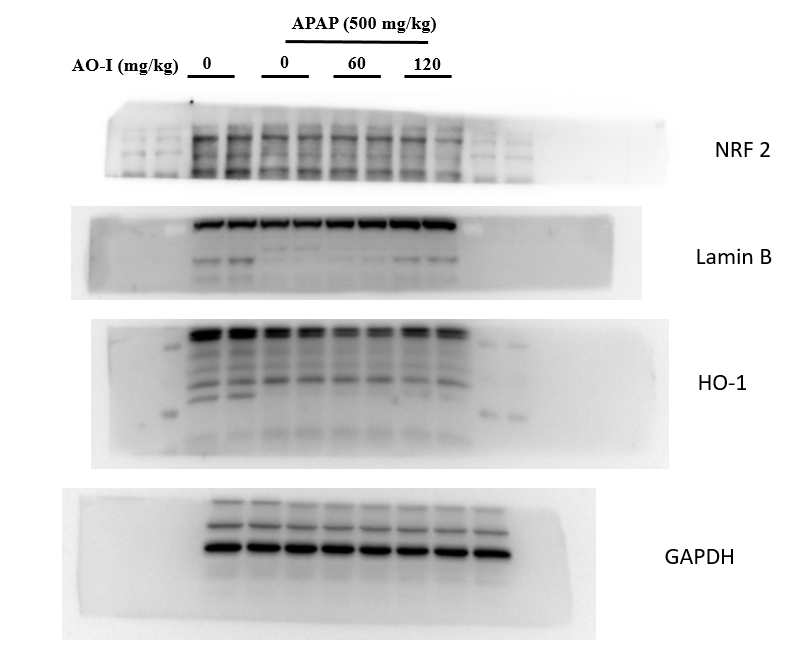

Supplement: Supplementary file 2 [file DataSheet2.DOCX]
